# Environment Knowledge-Driven Generic Models to Detect Coughs From Audio Recordings

**DOI:** 10.1109/OJEMB.2023.3271457

**Published:** 2023-04-28

**Authors:** Sudip Vhaduri, Sayanton V. Dibbo, Yugyeong Kim

**Affiliations:** Department of Computer and Information TechnologyPurdue University 311308 West Lafayette IN 47907 USA; Department of Computer ScienceDartmouth College 3728 Hanover NH 03755 USA; Field Service DepartmentLG Electronics Englewood Cliffs NJ 07632 USA

**Keywords:** Audio analytics, COPD, cough, COVID-19, microphone-sensing

## Abstract

*Goal:* Millions of people are dying due to respiratory diseases, such as COVID-19 and asthma, which are often characterized by some common symptoms, including coughing. Therefore, objective reporting of cough symptoms utilizing environment-adaptive machine-learning models with microphone sensing can directly contribute to respiratory disease diagnosis and patient care. *Methods:* In this work, we present three generic modeling approaches – *unguided*, *semi-guided*, and *guided* approaches considering three potential scenarios, i.e., when a user has no prior knowledge, some knowledge, and detailed knowledge about the environments, respectively. *Results:* From detailed analysis with three datasets, we find that *guided* models are up to 28% more accurate than the *unguided* models. We find reasonable performance when assessing the applicability of our models using three additional datasets, including two open-sourced cough datasets. *Conclusions:* Though *guided* models outperform other models, they require a better understanding of the environment.

## I. Introduction

### A. Motivation

According to the world health organization (WHO), over 6.5 million people have died worldwide since the outbreak in November 2019 [1]. COVID has become one of this century's most devastating respiratory diseases due to its high death toll and long-lasting health complexities. In addition to COVID-19, a range of inflammatory respiratory diseases, including chronic obstructive pulmonary disease (COPD), asthma, and many others, cause the magnitude of mortality and morbidity. According to the Centers for Disease Control and Prevention (CDC), annually, more than 15 million Americans are affected by COPD, and more than 150 thousand die of COPD each year, i.e., 1 death every 4 minutes due to COPD [2]. Due to asthma, on average, 10 Americans die daily, according to the Asthma and Allergy Foundation of America [3].

While these respiratory diseases are spreading human suffering and upending the lives of billions of people around the globe, they have some similarities in their symptoms. For example, common symptoms of COVID-19 are dry cough, fever, muscle or body aches, congestion, breathing difficulty, and fatigue, according to CDC [4]. Similarly, patients with COPD have coughing and difficulty in breathing [5]. Furthermore, asthma patients suffer from coughing and wheezing [6]. Thereby, coughing is found to be one of the major symptoms of several respiratory diseases, such as lung cancers, cystic fibrosis, aspiration, and bronchitis [7].

Therefore, a better and early understanding of cough and its patterns can help to assess people's condition and diagnosis of a disease, which is difficult in traditional approaches that rely on viral tests (based on samples from the nose and mouth) or antibody tests [8], chest X-ray or spirometry tests [9], blood tests, pulse oximetry, and sputum tests [10], [11] due to the time and resource requirements that are not available in most primary care access points or at homes. An automated and continuous reporting of cough symptoms using continuous smartphone-microphone sensing and predictive machine learning models can help us to overcome the limitations of current approaches. This smartphone-based objective cough reporting can help not only to detect people's conditions early but also can be very useful for monitoring patient conditions remotely. However, machine learning models are often trained in certain environments (e.g., clinics or homes) consisting of a known set of ambient sounds or noises [12], [13], [14] and may not generalize to new environments due to the lack of prior knowledge about the new backgrounds, i.e., unknown acoustical conditions or settings. For instance, models developed targeting forced coughs [15], [16] assume an ideal environment with low to no background noises, and models/apps developed for nocturnal cough detection [12], [17] assume relatively stable environments comprised of known continuous noises, such as air conditioner noises that do not change frequently compared to day time dynamic outdoor environments comprised of a wide range of known and unknown background noises. But incorporating prior knowledge about the environment is not always possible, especially for a new user. Therefore, designing a system that does not need prior knowledge about the background and can adapt over time will be helpful.

### B. Related Work

Researchers have been trying to objectively detect common symptoms of various **respiratory diseases**. A group of researchers has been using the wearable accelerator and stretchable strain sensors placed around the throat and on the chest to detect coughing as a symptom of different respiratory diseases [18]. They have been relying on an auto-encoder to classify cough with sensitivity and specificity. 92 and. 96 when a person is sitting. When a person is not sedentary or speaking, model performance may degrade. Also, sensors placed around the throat are not unobtrusive, and people may find it uncomfortable. Most importantly, wearable device user adherence drops over time [19], [20], [21], [22], [23], [24], [25], [26], [27], [28], [29], [30], [31], [32], [33], [34], [35], [36], [37], [38], [39].

Due to the aforementioned limitations, researchers have been using **audio recordings** to detect cough and different diseases [12], [13], [14], [29], [40], [41], [42]. For instance, one group of researchers has achieved up to. 88 sensitivity and. 99 specificity when detecting coughs using k-nearest-neighbor classifiers [40]. Furthermore, they have detected cough with. 93 sensitivity and. 89 specificity using SVM classifiers [41]. Similarly, another team of researchers has detected COVID-19 positive and non-COVID-19 coughs using a logistic regression classifier, a support vector machine classifier, and a gradient boosting classifier with precision and recall scores of around. 8 [42].

On the other hand, some researchers have used **deep neural network** models to detect coughs since they can be easily deployed on edge devices, including smartphones [15], [16], [43]. For instance, a team of researchers has detected bronchitis, bronchiolitis, pertussis, and healthy coughs using a convolutional neural network (CNN) with a precision score above. 8 [44]. Another group of researchers has detected COVID-19, pertussis, bronchitis, and healthy coughs using CNN and SVM classifiers with an overall accuracy of around. 88 [16]. However, the implementation has relied on two major components: (1) a smartphone app to record a user's forced coughs and (2) a cloud server to process and detect coughs from the smartphone audio recordings. Another team has detected symptomatic COVID-19 coughs and healthy coughs through a similar system consisting of deep neural networks and smartphone-server integration with an area under the receiver operating characteristic curve of. 88 [43]. These phone-server integration-based implementations have raised **privacy concerns** since privacy-sensitive raw audio data from a user's smartphone are sent to a remote cloud server.

To overcome privacy concerns, researchers have developed smartphone-based systems that do not require data upload [15]. However, this work still requires a user to cough in front of a smartphone microphone. These recordings are often captured in an idle environment with relatively lower background noise [15], [16]. Additionally, this approach may miss natural coughs (e.g., sleep time coughs), which can better represent a user's state and is also not applicable to dynamic environments with different background noises at varying intensities. Similarly, models/apps developed targeting nocturnal environments fail to work in dynamic daytime environments [12], [17]. Hence, there is a need for a generic cough detection system that can be used continuously and does not need prior knowledge about the environment, initially. Over time, the system can adapt as it gets a better understanding of the environment.

### C. Contribution

Adapted from our previous work [13], in this work, we present a trade-off between the availability of knowledge about a user's environment (i.e., a user's familiarity with the environment) and model performance, when identifying coughs utilizing three different modeling approaches, i.e., *unguided* (no prior knowledge is needed, but it is not the best performing model), *semi-guided* (some, but no specific prior knowledge is needed, resulting in a better-performing model than the *unguided* model), and *guided* (specific prior knowledge is needed, and it is the best performer) modeling approaches based on the availability of knowledge about the environments (Section II-B) to detect coughs from smartphone-microphone audio recordings. Compared to our previous work, in this work, our models are tailored to a user's knowledge about the surroundings. For example, an *unguided model* can be tailored to a user who has no prior knowledge about the background, *semi-guided* or *guided models* can be tailored to a user who has some knowledge about the surroundings. In this work, we utilize dynamic first and second temporal derivatives in addition to the *Mel-frequency cepstral coefficient* used in the previous work. In addition to the prior classifiers, we use the *gradient boosting*, which works better than other classifiers in most cases.

In this work, we test the applicability of our models using six distinct datasets, including two respiratory disease-specific datasets. The first three datasets (Sections II-C1–II-C3) are used to develop and determine the best models. To determine the applicability of generic cough models, we use three additional cough datasets (Sections II-C4–II-C6), including respiratory disease-specific COVID-19 and COPD datasets.

We find that the *guided* models can achieve around 12%–28% higher accuracy and $F_{1}$ score when compared to the *unguided* models (Sections III-A and III-B). Additionally, the *semi-guided* models perform relatively better than the *unguided* models. Therefore, *semi-guided* models can be an intermediate approach starting from the *unguided* and transitioning to the *guided* models for situations where a user does not have a clear idea about the environment at the beginning, but with the pass of time, the user can get a better understanding of environment-specific data.

## II. Materials and Methods

**Fig. 1.**
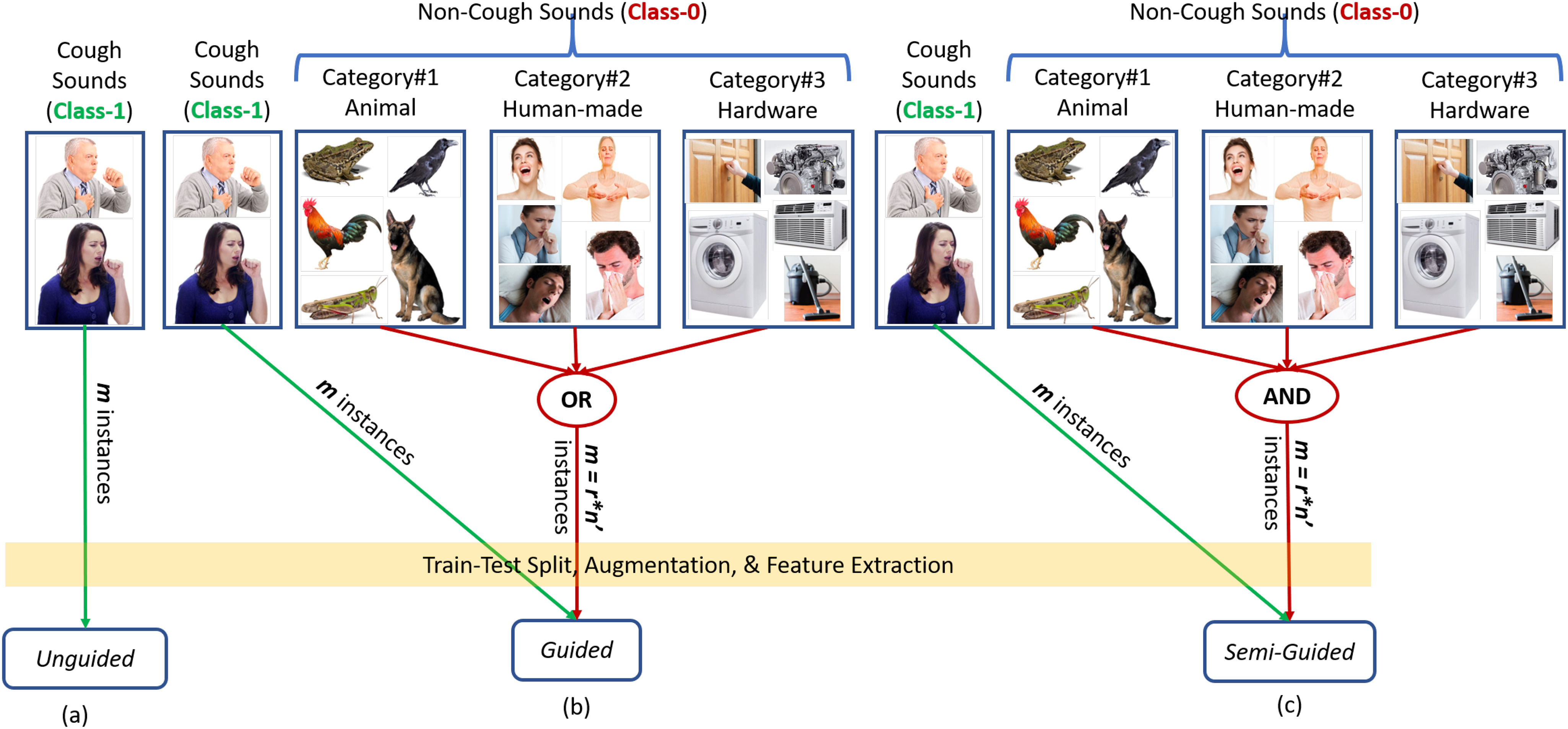
Knowledge-driven modeling schemes with cough sounds (class-1) and non-cough sound categories (class-0); $m$ stands for the total number of instances from the cough or non-cough class, $r$ ($= 5$ for *guided*, or 15 for *semi-guided*) stands for the total number of non-cough sound types used in class-0, and $n^{\prime }$ stands for the total number of instances per non-cough sound type (i.e., $n^{\prime } = m/r$) when modeling; Later, in Tables I and II, we present the values of these parameters when discussing our train-test methodology in Section II-D2.

While developing models to deploy in real-life settings, knowing the environments and the number of classes is always a major challenge. This problem is even more severe while developing models to detect a particular type of sound, e.g., cough, with or without the presence of various background noises in an unconstrained natural life. In Fig. 1, we present three modeling schemes with or without some knowledge about environments.

This section introduces three categories of non-cough sounds used in this work, followed by our three environment knowledge-based modeling approaches. Then, we introduce multiple datasets we utilize in this work, followed by our approaches to processing the data and developing models.

### A. Non-Cough Sound Categories

We utilize the following three categories of environmental sounds to construct our non-cough class (i.e., class-0).

• **Category#1 (Animal sounds)**: As a representation of animal sounds, we utilize five types of sounds, i.e., frog, crow, cricket, rooster, and dog sound recordings.
• **Category#2 (Human-made sounds)**: We use snoring, breathing, sneezing, laughing, and throat clearing (T/C) sound recordings as a representative of human-made sounds.
• **Category#3 (Hardware sounds)**: As a representation of hardware sounds, we include washing machine (W/D), door knock (D/K), vacuum cleaner (V/C), engine, and air conditioner (A/C) sound recordings.

### B. Knowledge-Driven Modeling Schemes

As depicted in Fig. 1, in this work, we present three modeling schemes based on a user's prior knowledge about the environments. Our modeling approaches are:

#### 1) Unguided Models

In this approach, we develop models assuming that a user does not have any prior knowledge about the environment composed of various sounds, except the target sound, e.g., cough (class-1). Therefore, we develop the unary (one class) models using only cough instances ($m$), as demonstrated in Fig. 1. In this unary modeling approach, part of the cough instances from class-1 will be used as non-cough instances depending on the values of the outlier threshold parameter, which will be presented in more detail in the “Parameter Optimization” section (Section II-E4). In the case of unary models, no non-cough instances will be used for model training. This will be further discussed in the “Training-Test Splits” section (Section II-D2). Though this type of model has broader applicability, it may underperform compared to the models developed with some prior knowledge about the environments.

#### 2) Guided Models

In this approach, we assume that a user has a detailed understanding of the environments and different noises in the backgrounds compared to *unguided* models. We develop three separate binary *guided* models considering one of the three background sound categories (Section II-A) as class-0 (non-cough class). Each sound category comprises five types of sounds (i.e., $r = 5$ as demonstrated in Fig. 1), and $n^{\prime } = m/r$ random non-cough instances will be picked from one of the five types of sounds uniformly for class balancing. This will be further discussed in the “Training-Test Splits” section (Section II-D2). In all cases, class-1 is composed of cough events. While it is expected that the binary models developed from one type of environment will work well in a similar type of environment, those models may struggle in other types of environments. For example, when models trained considering the presence of five types of animal sounds work well for similar backgrounds, they are expected to struggle when deploying/testing in environments with hardware noises.

#### 3) Semi-Guided Models

In this approach, we assume that a user has a better understanding of the background environment than in the case of the *unguided* models, but not as detailed as in the case of the *guided* models. Therefore, we utilize the coughs (class-1) and $r = 15$ types of non-cough sounds when developing binary models for the *semi-guided* environments (Fig. 1). For class balancing, $n^{\prime } = m/r$ random non-cough instances will be uniformly picked from the 15 sound types presented in Section II-A. This will be further discussed in the “Training-Test Splits” section (Section II-D2). The way these models are developed is expected to work better than the *unguided* models, but worse than the *guided* models.

In Section II-E2, we present the naming convention of different *unguided*, *guided*, and *semi-guided* models developed and tested in this work.

### C. Audio Datasets

In this manuscript, all our modeling and model performance assessments are based on six different audio datasets collected using smartphone microphones. To **develop models** **and determine the best models**, we utilize three datasets: 1) Environmental Sound Classification (abbreviated as ESC) dataset, 2) FreeSound dataset, and 3) Urban Sound 8 K (abbreviated as US-8 K) dataset. To **test the applicability of our models**, we use three additional datasets: 4) SoundSnap (abbreviated as SNP) dataset, 5) Coswara COVID-19 (abbreviated as COVID-19 or COVID) dataset, and 6) chronic obstructive pulmonary disease (abbreviated as COPD) dataset. When developing models, we also consider three categories of non-cough sounds to constitute class-0.

#### 1) ESC Dataset

To train-test different models, we utilize the Environmental Sound Classification (ESC) dataset [45], which is composed of 50 distinct sound types with 40 5-second-long labeled clips per type. The audio clips are recorded at 44.1 kHz frequency. We mainly use this dataset to obtain our training cough instances. Each audio recording is comprised of multiple two or three-phase cough events [13]. In addition to healthy people's cough sounds, in this work, we consider this dataset to obtain three categories of background sounds: 1) five types of **animal** sounds (i.e., frog, crow, cricket, rooster, and dog sounds), 2) four types of **human-made** sounds (i.e., snoring, breathing, sneezing, and laughing sounds), and 3) four types of **hardware** sounds (i.e., door knock (D/K), washing machine (W/D), vacuum cleaner (V/C), and engine sounds).

#### 2) FreeSound Dataset

We consider the FreeSound dataset [46] to obtain throat clearing (T/C) sounds as one of five types of **human-made** sounds used in this work. We obtained 37 clips that are $2.58 \pm 4.2$ seconds long and sampled at $44.6 \pm 4.2$ kHz frequency. For model development and noise augmentation, we use throat clearing (T/C) clips as common background noise. During binary-model training, these noise clips are used as part of class-0.

#### 3) US-8 K Dataset

To gather air conditioner sounds, i.e., one of the five types of **hardware** sounds used in this work, we utilize the Urban Sound 8 K (US-8 K) dataset [47], which is composed of 8732 labeled sound clips obtained from 10 urban sound types. Clips are up to 4-second and sampled at a frequency of 44.1 kHz. From this dataset, we consider 40 randomly picked air conditioner (A/C) sound clips as a source of common background sounds (class-0) while developing models.

#### 4) SNP Dataset

To determine the robustness of our models trained from ESC-coughs, we consider the SoundSnap (SNP) dataset [48] to obtain test cough sounds obtained from healthy people. Each audio clip consists of multiple cough events and is recorded at a sampling frequency of $46.65 \pm 11.10$ kHz. Therefore, we segment these cough clips into events (discussed in Section II-D1).

#### 5) COVID-19 Dataset

To determine the applicability of our models trained from the ESC-coughs, we use the cough recordings gathered from the Coswara COVID-19 dataset [49], [50]. The Coswara COVID-19 dataset is still growing up. Audio clips are recorded at a sampling rate of $47.82 \pm 0.83$ kHz. This dataset contains breathing, coughing, and speech sounds collected from healthy and unhealthy participants. We collect cough and breathing sounds from participants who tested positive for COVID-19. Throughout this manuscript, we interchangeably use the term “COVID” and “COVID-19” to indicate this dataset and the coughs obtained from the dataset.

#### 6) COPD Dataset

We also collect coughs from a set of 12 patients (average age of 56.2 $\pm$ 0.9 years) with chronic obstructive pulmonary disease and name the dataset as the COPD dataset [14]. We recorded coughs using the RecForge II smartphone application^1^^1^[Online]. Available: https://bit.ly/3TU3dBB at a sampling frequency of 44.1 kHz. We kept smartphones around one meter distant from the subjects. We utilize this COPD dataset to test the applicability of models developed from the ESC-coughs.

### D. Data Processing

Since we obtain data from various sources, we first modify the sampling frequency of all cough and non-cough audio events to a fixed sampling frequency of 44.1 kHz before any further processing. Next, we go through the following steps.

#### 1) Audio Segmentation and Cough Event Extraction

In this manuscript, we use various types of non-cough data that are already labeled. On the other hand, the cough sounds in a clip come with multiple cough events, either two or three phases [13]. Therefore, we follow a two-fold approach to collect cough event ground truths from audio clips. First, we use the Audacity desktop application [51] to load the audio clips and then perform a visual and auditory inspection to determine cough events and their phases before cropping/segmenting and storing. Next, we automate the process by developing an energy threshold-based audio segmentation followed by a phase classification approach, similar to the method developed in our previous work [12]. In Table I, we present a summary of cough events obtained from various datasets.

**TABLE I:** Summary of Cough Datasets

Dataset	purpose	# of	# of	duration
		coughs	subjects	(Sec)
		($m$)		
ESC	Train-Test	106	34	$0.36 \pm 0.14$
SNP	Test	106	40	$0.43 \pm 0.20$
COVID	Test	170	36	$0.81 \pm 0.93$
COPD	Test	282	12	$0.56 \pm 0.24$

#### 2) Training-Test Splits

For class balancing, we start with the same $m = 106$ instances from class-1 (cough class) and class-0 (non-cough class). As presented in Table II, for class-0, we uniformly pick the samples from the five types of sounds (i.e., $r = 5$ for one of the three *guided* models) or 15 types of sounds (i.e., $r = 15$ for the *semi-guided* models), gathered from the three sound categories. When splitting into train-test sets, we first randomly split the $m = 106$ original coughs 10 times using a 90%–10% mutually exclusive train-test split to perform 10 rounds of training and testing. This way, each split consists of around 96 (i.e., $\lceil 106*0.9\rceil$) train and 10 (i.e., 106 - 96 = 10) test coughs. Similarly, we pick the same number of random train-test non-cough instances uniformly from $r = 5$ (in case of each *guided* model) or $r = 15$ (in case of the *semi-guided* model) non-cough sound types. Thereby, we randomly pick $n^{\prime } = 21-22$, the number of instances from one of the three non-cough sound categories (**animal**, **human-made**, or **hardware**) consisting of $r = 5$ non-cough sound types as class-0 when developing *guided* models. Similarly, we randomly pick $n^{\prime } = 7-8$, the number of instances from the three non-cough sound categories (**animal**, **human-made**, and **hardware**) consisting of $r = 15$ non-cough sound types as class-0 when developing *semi-guided* models. In each split, we also consider the 17 augmentations (presented in the next section, i.e., Section II-D3) of each training cough event/instance in the training set. Similarly, we also consider the 17 augmentations of each test cough event/instance along with the original cough events/instances. Thereby, we obtain a total of 1728 (i.e., 96 * (1+17)) training instances and 180 (i.e., 10 * (1+17)) test instances from each class with mutual exclusion between train-test sets.

**TABLE II:** Summary of Non-Cough Sound Types; Symbols are Defined in Fig. 1 in Section II-B and “n/a” Stands for “not Available”

Non-	**Dataset:**	# of
cough	non-cough	instan-
sound	sound	ces per
types	types	type
(count, $r$)		($n^{\prime }$)
*Unguided* model		
n/a	-	0
($r = 0$)		
*Guided* model		
Animal	**ESC:** frog, crow, dog,	21-22
($r = 5$)	cricket, & rooster	
Human-	**ESC:** snoring, breathing,	21-22
made	sneezing, & laughing;	
($r = 5$)	**FreeSound:** throat clearing (T/C)	
Hardware	**ESC:** engine,	21-22
($r = 5$)	door knock (D/K),	
	vacuum cleaner (V/C), &	
	washing machine (W/D);	
	**US-8 K:** air conditioner (A/C)	
*Semi-guided* model		
Animal,	**ESC:** frog, crow, dog, cricket,	7-8
human-	rooster, snoring, breathing,	
made, &	sneezing, laughing,	
hardware	engine, D/K, V/C, & W/D;	
	**FreeSound:** throat clearing (T/C);	
($r = 15$)	**US-8 K:** air conditioner (A/C)	

#### 3) Data Augmentation

In real-world settings, audio cough recordings are altered due to variations in a user's physical and mental conditions (excitement, tiredness, exercise, and other numerous states) as well as the changes in the environments, i.e., backgrounds. To imitate these changes and capture the associated variations in audio recordings when developing models, we augment original cough and non-coughs events gathered from the US-8 K, FreeSound, and ESC datasets using various pitch shifts and time stretches. With these augmentations, we introduce data variation to train a model that is more resistant to overfitting. We use 14 pitch shifts ($\pm 0.5,\pm 1,\pm 1,5,\pm 2,\pm 2.5,\pm 3,\pm$3.5) and three time stretches (0.5, 0.25, and 0.75).

#### 4) Feature Extraction

In this work, we primarily use the *Mel-frequency cepstral coefficient* (MFCC) [52], which is a widely used method for spectral feature extraction when recognizing speech. In this feature extraction method, frequency bands are adapted to the human perception levels. However, using only MFCCs (static features) can flaw the locality. Therefore, we choose to use the first and second temporal derivatives ($\Delta$ and $\Delta -\Delta$) to mitigate the potential flaws of MFCCs. This combination of dynamic features and static MFCCs can be useful to increase the accuracy and the robustness of various audio event detection systems [53]. Thereby, we compute 40 MFCCs, 40 $\Delta$ and 40 $\Delta -\Delta$ features, i.e., a set of 120 candidate features from every cough and non-cough event.

### E. Model Development

In this section, we first present the classifiers and model naming conventions used in our modeling approach presented in this work. Next, we present the optimization steps.

#### 1) Classifiers

To develop binary models, we consider a set of classifiers, including the decision tree (abbreviated as DT), random forest (abbreviated as RF), naive bayes (abbreviated as NB), gradient boosting (abbreviated as GB), $k$-nearest neighbor (abbreviated as $k$-NN), and support vector machine (abbreviated as SVM) utilizing (1) polynomial kernel (Poly.) function (defined in Equation (1)) and (2) radial basis function (RBF) (defined in Equation (2)).

\begin{align*}
Poly. Kernel, K(y_{i},y_{j}) &= (1 + \gamma y_{i}^{T}y_{j})^{d} \tag{1}\\
RBF Kernel, K(y_{i},y_{j}) &= e^{-\gamma y_{i}^{T}y_{j}} \tag{2}
\end{align*}

Where $\gamma$ and $d$ are used to represent the “scale parameter” and “degree” parameters. In the equations, $y_{i}$ and $y_{j}$ are used to represent the two feature vectors. Also, we use parameter $C$ to indicate the misclassification penalty/cost. For unary models, we use the support vector machine with polynomial and RBF kernels supported by the Sci-kit learn machine learning package.

#### 2) Model Naming Convention

Throughout this manuscript, we follow a standard naming convention when referring to different models developed using different classifiers and sound categories. We use a compound term “Modeling approach – Classifier type”, followed by “Classifier abbreviation – Number of sound types used to make class-0”, followed by “ (sound category abbreviation)” to refer to a specific model. For example, “G-B RF-5 (M)” is used to indicate an optimal “*guided*” model trained with “binary random forest (RF)” classifier using the “five types of **human-made** sounds as class-0.” Similarly, “G-B GB-5 (A)” and “G-B RF-5 (H)” are used to indicate optimal “*guided*” models trained with “gradient boosting (GB)” and “random forest (RF)” classifiers using five types of background sounds gathered from the “**animal**” and “**hardware**” sounds, respectively, as class-0. Class-1 consists of coughs, as always. Similar to *guided* models, we use “S-B RF-15” to refer to an optimal “*semi-guided*” model trained with “binary random forest (RF)” classifier using fifteen types of sounds (gathered from the three sound categories) as class-0 and cough sounds as class-1. Since the negative class is comprised of all three sound categories, we simply drop the sound category from the term. Finally, we use “U-U SVM” to refer to an optimal “*unguided*” model trained with a “unary support vector machine (SVM)” classifier using only cough sounds. Since we do not have any non-cough sounds, we drop the class-0 constituting sound type count and sound categories from the term.

#### 3) Feature Optimization

We consider the “Select the $K$ Best” approach to determine the most dominant feature sets for binary classifier-based *guided* and *semi-guided* models. While training a model, we choose different sets of features and calculate the performance (ACC and $F_{1}$ scores) of the model using the 90% training data of a random split. We finally compute the average of 10 scores obtained from 10 separate splits for a specific type of model with a particular feature count. From our experiment, we find $K = 120$ is an optimal choice for the best *guided* and *semi-guided* models. Similarly, we consider a variance-based approach (i.e., smallest or largest variance) to select different sets of influential features for the unary classifier-based *unguided* models. From our experiment, we find 70 smallest variance feature is a good compromise for the best *unguided* models. In Table III, we present various classifiers/models with their optimal feature count.

**TABLE III:** Test Performance Summary (In Terms of Average $\pm$ Standard Deviation) of the *Unguided*, *Guided* and *Semi-Guided* Models When Tested in Environments With Three Categories of Non-Cough Sounds

Classifier (parameters with	Feature	ACC	FPR	FNR	$F_{1}$	AUC-	Preci-	Recall
optimal values)	count				score	ROC	sion	
***Unguided*****models** tested on								
ESC coughs and **Animal**								
SVM (Poly., $d=2$, $\nu$ =. 1)	70	.74$\pm$.04	. 4$\pm$.04	.11$\pm$.07	.77$\pm$.04		. 69$\pm$.02	. 89$\pm$.07
SVM (RBF, $\gamma =.0001$, $\nu$ =. 1)	10	. 63$\pm$.05	. 61$\pm$.05	. 13$\pm$.1	. 7$\pm$.05		. 59$\pm$.03	. 87$\pm$.1
ESC coughs and **Human-made**								
SVM (Poly., $d=2$, $\nu$ =. 1)	70	. 73$\pm$.04	. 43$\pm$.05	.11$\pm$.07	. 76$\pm$.04		. 67$\pm$.03	. 89$\pm$.07
SVM (RBF, $\gamma =.0001$, $\nu$ =. 1)	10	. 55$\pm$.04	. 77$\pm$.04	. 13$\pm$.1	. 66$\pm$.05		. 53$\pm$.03	. 87$\pm$.1
ESC coughs and **Hardware**								
SVM (Poly., $d=2$, $\nu$ =. 1)	70	. 85$\pm$.03	. 19$\pm$.04	. 11$\pm$.07	. 85$\pm$.03		. 83$\pm$.03	. 89$\pm$.07
SVM (RBF, $\gamma =.0001$, $\nu$ =. 1)	10	. 5$\pm$.05	. 87$\pm$.03	. 13$\pm$.1	. 63$\pm$.05		. 5$\pm$.03	. 87$\pm$.1
ESC coughs and all 3 categories								
SVM (Poly., $d=2$, $\nu$ =. 1)	70	. 76$\pm$.04	. 36$\pm$.04	. 11$\pm$.07	. 79$\pm$.04		0.71$\pm$.02	. 89$\pm$.07
SVM (RBF, $\gamma =.0001$, $\nu$ =. 1)	10	. 55$\pm$.05	. 77$\pm$.02	. 13$\pm$.1	. 66$\pm$.05		. 53$\pm$.03	. 87$\pm$.1
***Guided*****models** tested on								
ESC coughs and **Animal**								
DT (min_split=39,max_depth=10)	110	. 89$\pm$.03	. 06$\pm$.02	. 16$\pm$.06	. 88$\pm$.04	. 89$\pm$.03	. 93$\pm$.02	. 84$\pm$.06
NB (Bernoulli)	40	. 87$\pm$.03	. 12$\pm$.03	. 14$\pm$.05	. 87$\pm$.03	. 87$\pm$.02	. 87$\pm$.03	. 86$\pm$.05
RF (no_of_estimator=100)	100	. 94$\pm$.03	. 0$\pm$0	. 12$\pm$.06	. 93$\pm$.04	. 94$\pm$0	1$\pm$0	. 88$\pm$.06
SVM (RBF kernel,$\gamma$=.0001,C=1)	120	. 92$\pm$.03	. 01$\pm$0	. 15$\pm$.07	. 92$\pm$.04	. 92$\pm$.02	. 99$\pm$.01	. 85$\pm$.07
kNN (k= 2, Euclidean)	110	. 85$\pm$.05	0$\pm$0	. 31$\pm$.1	. 81$\pm$.07	. 85$\pm$.04	1$\pm$0	. 69$\pm$.1
SVM (Poly. kernel,d=2,C=1)	100	. 9$\pm$.04	. 02$\pm$.01	. 18$\pm$.08	. 89$\pm$.05	. 90$\pm$.04	. 97$\pm$.01	. 82$\pm$.08
GB (no_of_estimator=100)	120	. 95$\pm$.02	. 02$\pm$.01	. 08$\pm$.04	. 95$\pm$.02	. 95$\pm$0	. 98$\pm$.01	. 92$\pm$.04
ESC coughs and **Human-made**								
DT (min_split=39,max_depth=10)	120	. 8$\pm$.03	. 09$\pm$.02	. 31$\pm$.06	. 78$\pm$.04	. 80$\pm$.04	. 88$\pm$.03	. 69$\pm$.06
NB (Bernoulli)	30	. 82$\pm$.04	. 2$\pm$.02	. 16$\pm$.07	. 82$\pm$.04	. 82$\pm$.02	. 81$\pm$.02	. 84$\pm$.07
RF (no_of_estimator=100)	120	. 89$\pm$.04	. 02$\pm$.01	. 19$\pm$.08	. 88$\pm$.04	. 90$\pm$.01	. 97$\pm$.01	. 81$\pm$.08
SVM (RBF kernel,$\gamma$=.0001,C=1)	100	. 89$\pm$.05	. 02$\pm$.01	. 2$\pm$.11	. 87$\pm$.07	. 89$\pm$.03	. 97$\pm$.01	. 8$\pm$.11
kNN (k= 2, Euclidean)	80	. 81$\pm$.04	0$\pm$0	. 38$\pm$.08	. 77$\pm$.06	. 81$\pm$.04	1$\pm$0	. 62$\pm$.08
SVM (Poly. kernel,d=2,C=1)	100	. 84$\pm$.04	. 05$\pm$.02	. 28$\pm$.08	. 81$\pm$.06	. 84$\pm$.04	. 94$\pm$.02	. 72$\pm$.08
GB (no_of_estimator=100)	120	. 89$\pm$.05	. 05$\pm$.02	. 18$\pm$.1	. 87$\pm$.06	. 89$\pm$.02	. 95$\pm$.02	. 82$\pm$.1
ESC coughs and **Hardware**								
DT (min_split=39,max_depth=10)	50	. 87$\pm$.04	. 06$\pm$.02	. 2$\pm$.08	. 86$\pm$.05	. 87$\pm$.03	. 93$\pm$.02	. 8$\pm$.08
NB (Bernoulli)	110	. 93$\pm$.02	. 08$\pm$.01	. 06$\pm$.04	. 93$\pm$.02	. 93$\pm$.01	. 92$\pm$.01	. 94$\pm$.04
RF (no_of_estimator=100)	120	. 95$\pm$.02	. 02$\pm$.01	. 08$\pm$.04	. 95$\pm$.02	. 95$\pm$0	. 98$\pm$.01	. 92$\pm$.04
SVM (RBF kernel,$\gamma$=.0001,C=1)	110	. 92$\pm$.05	. 02$\pm$.01	. 14$\pm$.11	. 92$\pm$.06	. 92$\pm$.02	. 98$\pm$.01	. 86$\pm$.11
kNN (k= 2, Euclidean)	120	. 8$\pm$.06	0$\pm$0	. 4$\pm$.13	. 74$\pm$.11	. 80$\pm$.06	1$\pm$0	. 6$\pm$.13
SVM (Poly. kernel,d=2,C=1)	100	. 92$\pm$.03	. 01$\pm$.01	. 16$\pm$.07	. 91$\pm$.04	. 92$\pm$.01	. 99$\pm$.01	. 84$\pm$.07
GB (no_of_estimator=100)	120	. 95$\pm$.03	. 01$\pm$.01	. 09$\pm$.05	. 95$\pm$.03	. 95$\pm$.01	. 99$\pm$.01	. 91$\pm$.05
***Semi-guided*****models** tested on								
ESC coughs and all 3 categories								
DT (min_split=39,max_depth=10)	100	. 86$\pm$.04	. 08$\pm$.02	. 19$\pm$.07	. 85$\pm$.04	. 87$\pm$.05	. 91$\pm$.02	. 81$\pm$.07
NB (Bernoulli)	90	. 85$\pm$.04	. 15$\pm$.02	. 15$\pm$.08	. 85$\pm$.05	. 85$\pm$.03	. 85$\pm$.03	. 85$\pm$.08
RF (no_of_estimator=100)	120	. 94$\pm$.03	. 01$\pm$.01	. 1$\pm$.06	. 94$\pm$.03	. 95$\pm$.01	. 99$\pm$.01	. 9$\pm$.06
SVM (RBF kernel,$\gamma$=.0001,C=1)	120	. 93$\pm$.05	0$\pm$0	. 13$\pm$.1	. 93$\pm$.06	. 94$\pm$.01	1$\pm$0	. 87$\pm$.1
kNN (k= 2, Euclidean)	100	. 85$\pm$.05	0$\pm$0	. 31$\pm$.1	. 82$\pm$.07	. 85$\pm$.05	1$\pm$0	. 69$\pm$.1
SVM (Poly. kernel,d=2,C=1)	100	. 86$\pm$.04	. 01$\pm$.01	. 28$\pm$.07	. 83$\pm$.05	. 86$\pm$.04	. 99$\pm$.01	. 72$\pm$.07
GB (no_of_estimator=100)	120	. 93$\pm$.03	. 02$\pm$.01	. 13$\pm$0.07	. 92$\pm$.04	. 93$\pm$0	. 98$\pm$.01	. 87$\pm$.07

#### 4) Parameter Optimization

When training models with the 90% data, we utilize the grid search to determine optimal values for different parameters from a range of values, which includes degree, $d \in [1,3]$ with 1 increment (SVM Poly), $\gamma \in [.001,.01,.09]$ (SVM RBF), $C \in [1,3]$ with 1 increment (SVM), number of neighbors, $k \in [1,3]$ with 1 increment ($k$-NN), *Euclidean* distance function ($k$-NN), number_of_estimators $\in [100,300]$ with 100 increments (RF and GB), minimum split, min_split $\in [39,49]$ (DT), maximum depth, max_depth $\in [10,20]$ (DT), and outlier rate, $\nu \in \lbrace.0001,.001,.01,.1\rbrace$ (unary SVM) with 10 largest variance features. While training a model with 90% data of a split, we choose different sets of parameter values and calculate the performance (ACC and $F_{1}$ scores) of the model. We finally compute the average of 10 scores obtained from 10 separate splits for a specific type of model with a particular set of parameter values. In Table III, we present various classifiers/models with their associated set of parameters and their optimal values.

## III. Results

In this manuscript, we consider recall, accuracy (ACC), false positive rate (FPR), precision, false negative rate (FNR), and $F_{1}$ score to compare the performance of different modeling approaches. Additionally, consider the area under the curve-receiver operating characteristic (AUC-ROC) for the binary classifier-based models.

### A. Unguided Model Evaluation

As discussed in Section II-B1, we develop *unguided* models using unary SVM with “Polynomial kernel” (Poly.) (1) or “Radial Basis Function” (RBF) kernel (2). After training, we apply our trained models on the 10% test data (discussed in Section II-D2). In Table III, we summarize test performance values of different *unguided* models trained with the unary SVM classifiers utilizing only cough events. In the table, models are presented with their optimal parameter values and feature counts. We observe that the Poly. kernel-based *unguided* model (highlighted rows in the table) always outperforms the RBF kernel-based *unguided* model. While testing the *unguided* models in environments with the presence of 15 types of background sounds, we observe that the Poly. kernel-based model achieves 38% higher accuracy and $\approx$ 20% higher $F_{1}$ score than the RBF kernel-based model. Additionally, in the case of unary SVM Poly. kernel-based *guided* model, we observe on average $\approx$ 15% higher accuracy when testing in environments with **hardware** sounds compared to environments with **animal** or **human-made** sounds (Table III).

### B. Guided and Semi-Guided Model Evaluation

As discussed in Sections II-B2 and II-B3, we develop *guided* and *semi-guided* models using binary models. After training, we apply our trained models on the 10% test data (discussed in Section II-D2). In Table III, we summarize test performance of different *guided* and *semi-guided* models trained from cough events (class-1) and non-cough events (class-0). In the table, models are presented with their optimal parameter values and feature counts. When the gradient boosting (GB) *guided* model works the best for the environments that comprised of **animal** sounds (i.e., class-0), the random forest (RF) *guided* models work the best for the environments that comprised of **human-made** sounds and **hardware** sounds (i.e., class-0) (highlighted rows in the table). Among the three best *guided* environment models, we observe that the RF-based *guided* model for the environments with **human-made** has the lowest average accuracy of $.89 \pm. 04$. Compared to this human-made environment *guided* model, other two models achieve $\approx$ 7% (i.e., (.95-.89)/.89*100%, for both animal and hardware) higher accuracy. The lowest performance in environments with **human-made** background sounds can be explained by close similarity between coughs and other **human-made** sounds compared to **animal** and **hardware** background sounds.

In the case of *semi-guided* models, RF classifier-based model works the best in environments with the presence of all 15 types of background sounds (i.e., the last block of seven models in Table III). We also test the best *semi-guided* model, i.e., RF, on the three categories of environments separately and we obtain average accuracy of $.9 \pm. 02$ (animal), $.84 \pm. 03$ (human-made), and $.91 \pm. 03$ (hardware), respectively. This finding supports our previous findings while testing three separate *guided* models on their relevant environments.

**Comparison with the state-of-the-art:** We primarily use sensitivity (SEN = 1-FNR), specificity (SPE = 1-FPR), and AUC-ROC to compare the performance of our models with some benchmarks (Table IV). While our models can achieve the highest SEN and AUC-ROC compared to other works, our models suffer from lower SPE compared to others [40], [54], [55]. But we tested our models with a wide range of non-cough events compared to other works [40], [41], [54], [55]. Moreover, some work use ECG, thermistor, chest belt, accelerometer, and contact data in addition to audio data [55].

**TABLE IV:** Summary of the State-of-The-Art and Our Work

Ref.	SEN	SPE	AUC-	# of non-	# of
			ROC	cough types	data types
Our	. 98	. 92	. 95	15	1
[40]	. 88	. 99	. 93	3	1
[41]	. 92	. 88	. 90	6	1
[54]	. 95	. 94	. 94	3	1
[55]	. 94	. 94	. 94	4	7

### C. Model Comparison

In this section, we present the performance comparison among *unguided*, *semi-guided*, and *guided* models using different datasets. First, in Section III-C1, we present the model comparison when testing on the part of the three **known datasets** (i.e., ESC, FreeSound, and US-8 K datasets), but we keep the train and test sets mutually exclusive and change the background environments. In this comparison, we use the accuracy measure to compare different models when testing on various cough sounds (class-1) and non-cough sounds (class-0) separately.

Next, in Section III-C2, we compare the applicability of models (trained from the ESC, FreeSound, and US-8 K datasets) when testing on cough samples obtained from three **unknown datasets**, i.e., SNP, COPD, and COVID datasets. In this comparison, we primarily have test cough sounds (class-1) obtained from the unknown datasets. Therefore, we use the accuracy measure for performance comparison.

#### 1) Model Comparison Using Known Datasets With Varying Environments

**Fig. 2.**
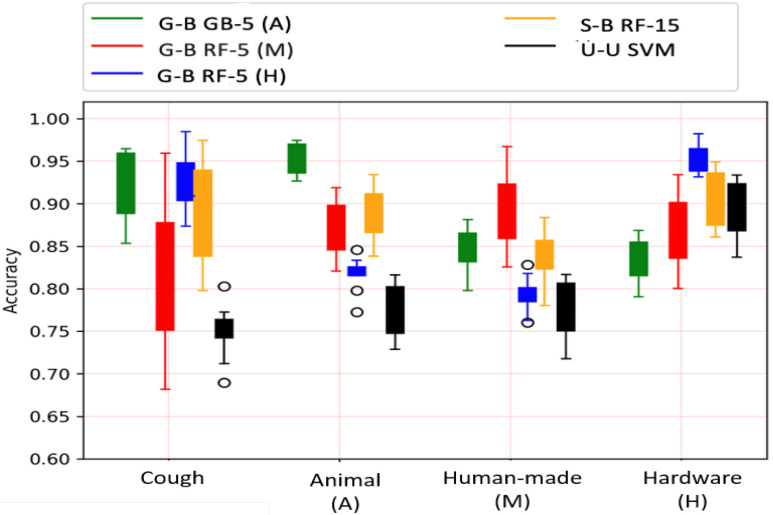
Model performance while testing on different sets.

In Fig. 2, we observe that, in general, *guided* and *semi-guided* models outperform the *unguided* model. When testing three types of models on cough sounds we observe that *guided* models, except the “G-B RF-5 (M)”, perform better than the *semi-guided* model, which outperforms the *unguided* model, i.e., “U-U SVM model.” Similarly, when comparing the three *guided* models, we observe that models trained and tested in similar environments outperform the other two models trained from different environments. For example, when testing on the five types of **animal** sounds, the “G-B GB-5 (A)” *guided* model outperforms the other two *guided* models trained from **human-made** sounds (i.e., “G-B RF-5 (M)”) and **hardware** sounds (i.e., “G-B RF-5 (H)”). Similarly, the “G-B RF-5 (M)” *guided* model works the best when tested on **human-made** sounds and the “G-B RF-5 (H)” *guided* model perform the best when tested on **hardware** sounds. Compared to animal and hardware sounds, human-made sounds lead to lower performance while applying the best *guided* models on their relevant sounds/environments. This is similar to what we have observed and discussed in Section III-B.

In the upper part of Table V, we summarize the test accuracy values of various models using different datasets. In general, we observe improvements when moving from *unguided* to *semi-guided* to *guided* models. Compared to the *unguided* models, we achieve an increase in average accuracy by 18% (cough), 22% (animal), 14% (human-made), and 7% (hardware) using the best *guided* models, decided based on the highest confidence. All three types of models down perform when applying on **human-made** sounds.

**TABLE V:** Model Test Performance Comparison

Comparison with **known datasets** (Section III-C1)			
Average accuracy across different sound categories			
Type/Category	*Unguided*	*Semi-Guided*	Best *Guided*
Cough (ESC)	. 78$\pm$.03	. 89$\pm$.06	. 92$\pm$.04
Animal	. 78$\pm$.03	. 89$\pm$.03	. 95$\pm$.02
Human-made	. 78$\pm$.04	. 84$\pm$.03	. 89$\pm$.04
Hardware	. 89$\pm$.04	. 91$\pm$.03	. 95$\pm$.02
Comparison with **unknown datasets** (Section III-C2)			
Average accuracy across different cough datasets			
Dataset	*Unguided*	*Semi-Guided*	Best *Guided*
ESC	. 92$\pm$.02	1$\pm$0	1$\pm$0
SNP	. 92$\pm$.01	. 91$\pm$.02	. 96$\pm$.01
COVID	. 92$\pm$0	. 92$\pm$.02	. 98$\pm$0
COPD	. 9$\pm$.01	. 74$\pm$.01	. 92$\pm$.01

**Fig. 3.**
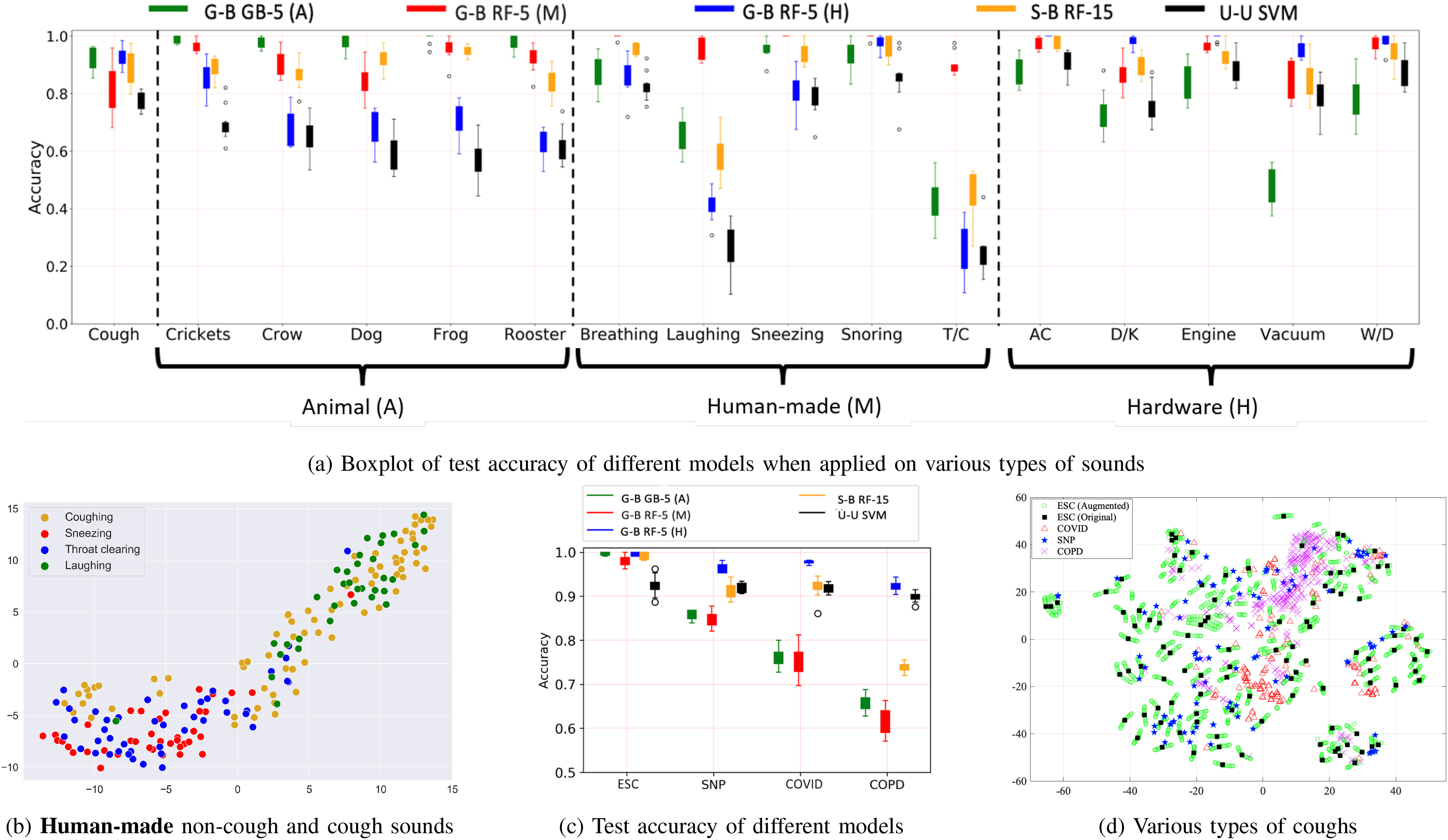
(a) and (c) boxplot of model performance; (b) and (d) t-SNE plot of different data distribution.

Next, we further investigate the detailed performance of three types of models while testing on one of the five types of sounds within each category. In Fig. 3(a), we summarize the performance values of different models utilizing boxplots. In general, *unguided* models perform the worst among the three types of models, *guided* models with similar backgrounds perform the best, and among the three *guided* models **human-made** sound data-driven models perform the worst in the case of individual sound types. These findings are very similar to what we have observed so far in the case of aggregated analysis. Additionally, in Fig. 3(a), we find that compared to *unguided* models, *guided* models are, on average, around 65% (when tested on laughing and throat-clearing (T/C) sounds) and 20% (when tested on breathing, sneezing, and snoring) more accurate. That is, the difference between the *unguided* and the best *guided* model is huge when tested on laughing and throat clearing (T/C), compared to the remaining three **human-made** sounds (i.e., breathing, sneezing, and snoring).

Next, we investigate the low performance of all models when testing on the “laughing” and “throat clearing” (T/C) sounds (observed in Fig. 3(a)). In Fig. 3(b), we use the t-distributed stochastic neighbor embedding (t-SNE) plot, which is a way to explore the relationship among high-dimensional neighbors in a two-dimensional plane, to compare the data distribution of the “laughing”, “throat clearing” (T/C), and “cough” sounds. We also plot the t-SNE distribution of “sneezing” sounds (one of the sounds types where models achieve high accuracy) to better understand the issues that lead to low performance when classifying the “laughing” and “throat clearing” sounds compared to other **human-made** sounds, such as “sneezing.” In the figure, every data sample is a two-dimensional representation of the 120 features (obtained from the three sets of features, i.e., MFCCs, $\Delta$, and $\Delta -\Delta$) of that sample. We obverse that “laughing” and “throat clearing” sounds are overlapped with “cough” sounds compared to the “sneezing” sounds; thereby, classification models find it more challenging to distinguish “laughing” or “throat clearing” sounds from “cough” sounds compared to other **human-made** sounds, such as “sneezing.”

#### 2) Model Applicability Comparison Using Unknown Datasets

In the lower part of Table V, we find that *guided* models achieve relatively higher accuracy compared to the *unguided* and *semi-guided* models as before. *Guided* models achieve the highest average accuracy of 1.0 for the ESC coughs (with 10 random 90%–10% train-test splits) and the lowest average accuracy of. 92 for COPD coughs. Furthermore, these *guided* models achieve at least. 96 average accuracy when tested on SNP or COVID cough datasets.

In Fig. 3(c), we demonstrate more detailed analysis of model performances using boxplots when testing the three types of ESC-cough trained models (i.e., *unguided*, *semi-guided*, and *guided* models) on various cough datasets. We find that “G-B RF-5 (H)” models, i.e., *guided* models developed using the ESC-coughs as class-1 and **hardware** sounds as class-0, achieve higher accuracy than the other two types of *guided* models trained with class-0 comprised of **animal** sounds or **human-made** sounds, i.e., “G-B GB-5 (A)” and “G-B RF-5 (M)” models in general. In the figure, we find that “G-B RF-5 (H)” models, i.e., *guided* models developed with class-0 comprised of **hardware** environmental sounds, consistently achieve more than. 95 accuracy across all types of coughs, except the COPD coughs, where the models achieve more than. 9 accuracy. On the other hand, *guided* models developed using class-0 comprised of **animal** sounds or **human-made** sounds are less accurate, i.e., lower than. 75, when applying on COPD or COVID cough datasets.

Next, we utilize the t-SNE plot to investigate model performance variation across various cough datasets. In Fig. 3(d), we present the distribution of various coughs gathered from all four cough datasets, i.e., SNP (blue pentagons), ESC, COPD (pink crosses), and COVID (red triangles). When assessing model test performance, we use the original cough events obtained from the SNP, COPD, and COVID datasets. However, we use both original and augmented (17 augmentations discussed in Section II-D3) ESC coughs to train-test models using 90%–10% splits. Therefore, in the t-SNE plot, we consider both the original (black squares) and augmented (green circles) versions of ESC coughs, but the original coughs from SNP, COPD, and COVID datasets.

In Fig. 3(d), we find that SNP cough instances (blue pentagons) and ESC cough instances (black squares and green circles) are completely overlapped. Therefore, models developed with ESC coughs can easily identify SNP coughs. However, the COVID cough instances (red triangles) and COPD cough instances (pink crosses) create two clusters that are separable from the ESC coughs. Thereby, ESC-cough trained models struggle to identify COPD and COVID cough instances. Compared to the COVID cluster, the COPD cluster is composed of more instances. Therefore, ESC-cough trained models underperform when applied to identify COPD coughs compared to COVID coughs.

## IV. Discussion

In this work, we attempt to develop three types of generic cough models based on a user's prior knowledge about the surrounding environment and try to detect different types of coughs, including coughs obtained from patients with two respiratory diseases (COVID-19 and COPD). We find that a user can expect to get better performance (ACC or $F_{1}$ score) when identifying cough and non-cough sounds utilizing the best *guided* models compared to the *unguided* models. But, the *guided* models require a user to have a better understanding of the environment compared to the *unguided* models, where a user does not need prior knowledge about the surroundings. We also find that *semi-guided* models perform relatively better than the *unguided* models. Thereby, when a user does not have any idea about the environment, the user can start with the *unguided* models. As time passes and the user has some idea about the environment, *semi-guided* models can replace the *unguided* models. Finally, when the user has a clear idea about the environment, *guided* models can replace the *semi-guided* models to provide a highly accurate decision.

We find that ESC cough-trained generic *unguided*, *semi-guided*, and the best *guided* achieve consistent accuracy across unknown datasets, except the COPD dataset (lower part of Table V). Therefore, disease-specific models can be developed to detect chronic coughs, such as COPD. However, in the case of a sudden pandemic outbreak, such as COVID-19, it is difficult to find enough data from patients to train disease-specific models during the early stage of the outbreaks. In such cases, we can start with generic cough models, and over time, we can develop mixed models from the generic cough models using transfer learning. Mixed models will require relatively fewer disease-specific coughs than the disease-specific models trained from more extensive disease-specific data.

A major limitation of our work is the limited number of cough and non-cough events and the unavailability of different non-cough human sounds obtained from patients in unknown datasets. However, we augment the original cough sounds to create the effect of changes in the natural environment and a user's physical condition or mental state, and randomly split the entire dataset 10 times when developing models to circumvent various issues, including overfitting and data sparsity. Therefore, our findings show a promise, which can further be investigated and validated with a large-scale extended period longitudinal study with varying diseases, patient demographic, types of non-cough human sounds, and advanced models.

Furthermore, the drop in performance when testing healthy people's cough models on patients could be due to differences in voiced phases (e.g., frequency and noise) between coughs from patients and healthy people. Also, the voiced phase does not always appear and may get confused with some parts of laughing or throat clearing. Confounding factors, such as device variability, may affect data distribution (e.g., Fig. 3(d)) and model performance. Also, in real-world deployment, model performance can be affected by device positioning and placement. To overcome the barrier effect some standard techniques can be adopted [56], [57], [58], [59]. All of these will require detailed investigation and beyond the scope of this work. Additionally, in a real-world deployment, as the system make a transition from *unguided* to *semi-guided* or *guided* models with time pass, the system can identify different background sounds using sound classification approaches [60], [61], [62] and retrain the initial *unguided* cough model to obtain more robust models utilizing relevant background sounds by following approaches similar to the Federated learning, environment knowledge broadcast among users, and place discovery [63], [64], [65], [66], [67], [68]. These are beyond the scope of this manuscript.

While our findings in this work show the promise of developing models to detect cough symptoms utilizing a user's background environment knowledge about the presence of different types of sounds, the effective applicability of such models for disease diagnosis depends on many other factors, including detecting other symptoms (e.g., breathing difficulty) and integration of the self-reported subjective symptoms in addition to objective predictions [69], [70], [71], [72], [73], [74], [75]. Additionally, people's medical history and health records can be integrated for better diagnosis of diseases and people's conditions. This will require careful investigation with additional large-scale longitudinal studies with diverse subject groups and diseases.

## V. Conclusion

When evaluating our modeling approaches (i.e., *unguided, semi-guided, and guided* modeling approaches) using 10 random splits, we find that a user can expect to get 12%–28% higher accuracy and $F_{1}$ score when identifying cough and non-cough sounds utilizing *guided* models compared to the *unguided* models (Table III). We also find that *semi-guided* models outperform the *unguided* models. While this work shows the feasibility of the approach, additional studies will be required for the clinical validation of models before commercializing the work.

## References

[1] “WHO coronavirus disease (COVID-19) dashboard.” 2023. Accessed: Apr. 20, 2023. [Online]. Available: https://covid19.who.int/

[2] “COPD–Key Facts.” 2022. Accessed: Apr. 20, 2023. [Online]. Available: https://bit.ly/3MLzerS

[3] “Asthma facts and figures.” 2023. Accessed: Apr. 20, 2023. [Online]. Available: https://bit.ly/3KzX08f

[4] “CDC: Symptoms of COVID-19.” 2022. Accessed: Apr. 20, 2023. [Online]. Available: https://bit.ly/3OXD5Tm

[5] C. Clinic, “Controlled coughing for COPD patients.” 2023. Accessed: Apr. 20, 2023. [Online]. Available: https://cle.clinic/3tEDlx4

[6] ”Asthma - symptoms and causes - mayo clinic.” 2022. Accessed: Apr. 20, 2023. [Online]. Available: https://mayocl.in/3vmT3zc

[7] C. B. Simpson and M. R. Amin, “Chronic cough: State-of-the-art review,” Otolaryngology–Head Neck Surg., vol. 21, pp. 693–700, 2006. 10.1016/j.otohns.2005.11.014 16564398

[8] “CDC: COVID-19 testing.” 2022. Accessed: Apr. 20, 2023. [Online]. Available: https://bit.ly/3nRjYOM

[9] “COPD symptoms and diagnosis| American lung association.” 2023. Accessed: Apr. 20, 2023. [Online]. Available: https://bit.ly/3hefi2f

[10] “Pneumonia | Disease or condition of the week| CDC”. 2021. Accessed: Apr. 20, 2023. [Online]. Available: https://bit.ly/35aOE7Q

[11] “Asthma: Steps in testing and diagnosis - mayo clinic.” 2022. Accessed: Apr. 20, 2023. [Online]. Available: https://mayocl.in/3vPs3J7

[12] S. Vhaduri, T. V. Kessel, B. Ko, D. Wood, S. Wang, and T. Brunschwiler, “Nocturnal cough and snore detection in noisy environments using smartphone-microphones,” in Proc. IEEE Int. Conf. Healthcare Inform., 2019, pp. 1–7.

[13] S. Vhaduri, “Nocturnal cough and snore detection using smartphones in presence of multiple background-noises,” in Proc. 3rd ACM SIGCAS Conf. Comput. Sustain. Societies, 2020, pp. 174–186.

[14] S. V. Dibbo, Y. Kim, and S. Vhaduri, “Effect of noise on generic cough models,” in Proc. IEEE 17th Int. Conf. Wearable Implantable Body Sensor Netw., 2021, pp. 1–4.

[15] J. Laguarta, F. Hueto, and B. Subirana, “COVID-19 artificial intelligence diagnosis using only cough recordings,” IEEE Open J. Eng. Med. Biol., vol. 1, pp. 275–281, 2020. 34812418 10.1109/OJEMB.2020.3026928 PMC8545024

[16] A. Imran , “Ai4COVID-19: Ai enabled preliminary diagnosis for COVID-19 from cough samples via an app,” Inform. Med. Unlocked, vol. 20, 2020, Art. no. 100378. 10.1016/j.imu.2020.100378 PMC7318970 32839734

[17] “myCough,” Accessed: Apr. 20, 2023. [Online]. Available: https://www.mycough.ch

[18] T. Otoshi , “A novel automatic cough frequency monitoring system combining a triaxial accelerometer and a stretchable strain sensor,” Sci. Rep., vol. 11, no. 1, pp. 1–9, 2021. 33976286 10.1038/s41598-021-89457-0 PMC8113562

[19] A. Lazar, C. Koehler, J. Tanenbaum, and D. H. Nguyen, “Why we use and abandon smart devices,” in Proc. ACM Int. Joint Conf. Pervasive Ubiquitous Comput., 2015, pp. 635–646.

[20] S. Vhaduri and C. Poellabauer, “Biometric-based wearable user authentication during sedentary and non-sedentary periods,” in Proc. Int. Workshop Secur. Privacy Internet-of-Things, 2018, pp. 1–4.

[21] W. Cheung and S. Vhaduri, “Continuous authentication of wearable device users from heart rate, gait, and breathing data,” in Proc. IEEE 8th RAS EMBS Int. Conf. Biomed. Robot. Biomechatronics, 2020, pp. 587–592.

[22] W. Cheung and S. Vhaduri, “Context-dependent implicit authentication for wearable device users,” in Proc. IEEE 31st Int. Symp. Personal, Indoor Mobile Radio Commun., 2020, pp. 1–7.

[23] A. Muratyan, W. Cheung, S. V. Dibbo, and S. Vhaduri, “Opportunistic multi-modal user authentication for health-tracking IoT wearables,” in Proc. EAI 5th Int. Conf. Saf. Secur. Internet Things, 2021, pp. 1–18.

[24] S. Vhaduri, S. V. Dibbo, and W. Cheung, “HIAuth: A hierarchical implicit authentication system for IoT wearables using multiple biometrics,” IEEE Access, vol. 9, pp. 116395–116406, 2021.

[25] S. Vhaduri, S. V. Dibbo, and C.-Y. Chen, “Predicting a user's demographic identity from leaked samples of health-tracking wearables and understanding associated risks,” in Proc. IEEE 10th Int. Conf. Healthcare Inform., 2022, pp. 309–318.

[26] S. Vhaduri and C. Poellabauer, “Multi-modal biometric-based implicit authentication of wearable device users,” IEEE Trans. Inf. Forensics Secur., vol. 14, no. 12, pp. 3116–3125, Dec. 2019.

[27] S. Vhaduri and C. Poellabauer, “Wearable device user authentication using physiological and behavioral metrics,” in Proc. IEEE 28th Int. Symp. Pers., Indoor, Mobile Radio Commun., 2017, pp. 1–6.

[28] S. Vhaduri and C. Poellabauer, “Impact of different pre-sleep phone use patterns on sleep quality,” in Proc. IEEE 15th Int. Conf. Wearable Implantable Body Sensor Netw., 2018, pp. 94–97.

[29] S. Vhaduri and T. Brunschwiler, “Towards automatic cough and snore detection,” in Proc. IEEE Int. Conf. Healthcare Inform., 2019, p. 1.

[30] C.-Y. Chen, S. Vhaduri, and C. Poellabauer, “Estimating sleep duration from temporal factors, daily activities, and smartphone use,” in Proc. IEEE Annu. 44th Comput. Soc. Computers, Software, Appl. Conf., 2020, pp. 545–554.

[31] S. Vhaduri and T. Prioleau, “Adherence to personal health devices: A case study in diabetes management,” in Proc. EAI 14th Int. Conf. Pervasive Comput. Technol. Healthcare, 2020, pp. 62–72.

[32] S. V. Dibbo, W. Cheung, and S. Vhaduri, “On-phone CNN model-based implicit authentication to secure IoT wearables,” in Proc. EAI 5th Int. Conf. Saf. Secur. Internet Things, 2021, pp. 19–34.

[33] S. Vhaduri, S. V. Dibbo, C.-Y. Chen, and C. Poellabauer, “Predicting next call duration: A future direction to promote mental health in the age of lockdown,” in Proc. IEEE 45th Annu. Comput. Softw. Appl. Conf., 2021, pp. 804–811.

[34] Y. Kim, S. Vhaduri, and C. Poellabauer, “Understanding college students' phone call behaviors towards a sustainable mobile health and wellbeing solution,” in Proc. Int. Conf. Syst. Eng., 2020, pp. 1–6.

[35] S. Vhaduri, S. V. Dibbo, and Y. Kim, “Deriving college students' phone call patterns to improve student life,” IEEE Access, vol. 9, pp. 96453–96465, 2021.

[36] S. Vhaduri and C. Poellabauer, “Summary: Multi-modal biometric-based implicit authentication of wearable device users,” IEEE Trans. Inf. Forensics Secur., vol. 14, no. 12, pp. 3116–3125, Dec. 2019.

[37] S. V. Dibbo, Y. Kim, S. Vhaduri, and C. Poellabauer, “Visualizing college students' geo-temporal context-varying significant phone call patterns,” in Proc. IEEE 9th Int. Conf. Healthcare Inform., 2021, pp. 381–385.

[38] S. Vhaduri and S. Simhadri, “Understanding user concerns and choice of app architectures in designing audio-based mHealth apps,” Elsevier Smart Health J., vol. 26, 2022, Art. no. 100341.

[39] S. Vhaduri, W. Cheung, and S. V. Dibbo, “Bag of on-phone ANNs to secure IoT objects using wearable and smartphone biometrics,” IEEE Trans. Dependable Secure Comput., early access, Apr. 21, 2023, doi: 10.1109/TDSC.2023.3269037.

[40] J. Monge-Álvarez, C. Hoyos-Barceló, P. Lesso, and P. Casaseca-de-la-Higuera, “Robust detection of audio-cough events using local hu moments,” IEEE J. Biomed. Health Inform., vol. 23, no. 1, pp. 184–196, Jan. 2019. 29994432 10.1109/JBHI.2018.2800741

[41] J. Monge-Álvarez, C. Hoyos-Barceló, L. M. San-José-Revuelta, and P. Casaseca-de-la-Higuera, “A machine hearing system for robust cough detection based on a high-level representation of band-specific audio features,” IEEE Trans. Biomed. Eng., vol. 66, no. 8, pp. 2319–2330, Aug. 2019. 30575527 10.1109/TBME.2018.2888998

[42] C. Brown , “Exploring automatic diagnosis of COVID-19 from crowdsourced respiratory sound data,” in Proc. ACM SIGKDD Int. Conf. Knowl. Discov. Data Mining, 2020, pp. 3474–3484.

[43] H.-A. Rashid, M. M. Sajadi, and T. Mohsenin, “CoughNet-V2: A scalable multimodal DNN framework for point-of-care edge devices to detect symptomatic COVID-19 cough,” in Proc. IEEE Healthcare Innov. Point Care Technol., 2022, pp. 37–40.

[44] C. Bales , “Can machine learning be used to recognize and diagnose coughs?,” in Proc. IEEE Int. Conf. e-Health Bioeng., 2020, pp. 1–4.

[45] K. J. Piczak, “Esc: Dataset for environmental sound classification,” in Proc. 23rd ACM Int. Conf. Multimedia, 2015, pp. 1015–1018.

[46] “FreeSound.” 2023. Accessed: Apr. 20, 2023. [Online]. Available: https://freesound.org/

[47] “Urbansound8k dataset.” 2005. Accessed: Apr. 20, 2023. [Online]. Available: https://bit.ly/2uHhhYh

[48] “Find the perfect sound effect.” 2023. Accessed: Apr. 20, 2023. [Online]. Available: https://www.soundsnap.com

[49] “Coswara-data.” 2020. Accessed: Apr. 20, 2023. [Online]. Available: https://github.com/iiscleap/Coswara-Data

[50] N. Sharma , “Coswara–a database of breathing, cough, and voice sounds for COVID-19 diagnosis,” in Proc. Annu. Conf. Int. Speech Commun. Assoc., INTERSPEECH, vol. 2020, 2020, pp. 4811–4815.

[51] “Audacity: Free, open source, cross-platform audio software.” 2023. Accessed: Apr. 20, 2023. [Online]. Available: https://www.audacityteam.org/

[52] S. G. Koolagudi, D. Rastogi, and K. S. Rao, “Identification of language using mel-frequency cepstral coefficients (MFCC),” Procedia Eng., vol. 38, pp. 3391–3398, 2012.

[53] K. Kumar, C. Kim, and R. M. Stern, “Delta-spectral cepstral coefficients for robust speech recognition,” in Proc. IEEE Int. Conf. Acoust. Speech Signal Process., 2011, pp. 4784–4787.

[54] T. Drugman, J. Urbain, and T. Dutoit, “Assessment of audio features for automatic cough detection,” in Proc. IEEE 19th Eur. Signal Process. Conf., 2011, pp. 1289–1293.

[55] T. Drugman , “Objective study of sensor relevance for automatic cough detection,” IEEE J. Biomed. Health Inform., vol. 17, no. 3, pp. 699–707, May 2013. 24592470 10.1109/jbhi.2013.2239303

[56] M. Green and D. Murphy, “Environmental sound monitoring using machine learning on mobile devices,” Appl. Acoust., vol. 159, 2020, Art. no. 107041.

[57] K. Doddabasappla and R. Vyas, “Statistical and machine learning-based recognition of coughing events using triaxial accelerometer sensor data from multiple wearable points,” IEEE Sens. Lett., vol. 5, no. 6, pp. 1–4, Jun. 2021.36789370

[58] X. Sun, Z. Lu, W. Hu, and G. Cao, “SymDetector: Detecting sound-related respiratory symptoms using smartphones,” in Proc. ACM Int. Joint Conf. Pervasive Ubiquitous Comput., 2015, pp. 97–108.

[59] L. Cesnakova , “Continuous sound collection using smartphones and machine learning to measure cough,” Digit. Biomarkers, vol. 3, no. 3, pp. 166–175, 2019. 10.1159/000504666 PMC7011715 32095775

[60] J. Singh and R. Joshi, “Background sound classification in speech audio segments,” in Proc. IEEE Int. Conf. Speech Technol. Hum.- Comput. Dialogue, 2019, pp. 1–6.

[61] J. Salamon and J. P. Bello, “Deep convolutional neural networks and data augmentation for environmental sound classification,” IEEE Signal Process. Lett., vol. 24, no. 3, pp. 279–283, Mar. 2017.

[62] A. Dayal , “Lightweight deep convolutional neural network for background sound classification in speech signals,” J. Acoustical Soc. Amer., vol. 151, no. 4, pp. 2773–2786, 2022. 10.1121/10.0010257 35461490

[63] S. Vhaduri and C. Poellabauer, “Cooperative discovery of personal places from location traces,” in Proc. IEEE 25th Int. Conf. Comput. Commun. Netw., 2016, pp. 1–9.

[64] S. Vhaduri and C. Poellabauer, “Hierarchical cooperative discovery of personal places from location traces,” IEEE Trans. Mobile Comput., vol. 17, no. 8, pp. 1865–1878, Aug. 2018.

[65] S. Vhaduri, C. Poellabauer, A. Striegel, O. Lizardo, and D. Hachen, “Discovering places of interest using sensor data from smartphones and wearables,” in Proc. IEEE Ubiquitous Intell. Comput., 2017, pp. 1–8.

[66] S. Vhaduri and C. Poellabauer, “Opportunistic discovery of personal places using smartphone and fitness tracker data,” in Proc. IEEE Int. Conf. Healthcare Inform., 2018, pp. 103–114.

[67] S. Vhaduri and C. Poellabauer, “Opportunistic discovery of personal places using multi-source sensor data,” IEEE Trans. Big Data, vol. 7, no. 2, pp. 383–396, Jun. 2021.

[68] M. T. Al Amin, S. Barua, S. Vhaduri, and A. Rahman, “Load aware broadcast in mobile ad hoc networks,” in Proc. IEEE Int. Conf. Commun., 2009, pp. 1–5.

[69] H. Coppock , “Audio-based AI classifiers show no evidence of improved COVID-19 screening over simple symptoms checkers,” 2022, *arXiv:2212.08570*.

[70] S. Vhaduri and C. Poellabauer, “Human factors in the design of longitudinal smartphone-based wellness surveys,” in Proc. IEEE Int. Conf. Healthcare Inform., 2016, pp. 156–167. 10.1007/s41666-017-0003-8 PMC8981805 35415393

[71] S. Vhaduri, A. Munch, and C. Poellabauer, “Assessing health trends of college students using smartphones,” in Proc. IEEE Healthcare Innov. Point-of-Care Technol. Conf., 2016, pp. 70–73.

[72] S. Vhaduri and C. Poellabauer, “Design and implementation of a remotely configurable and manageable well-being study,” in Proc. Int. Summit Smart City 360○, 2015, pp. 179–191.

[73] S. Vhaduri and C. Poellabauer, “Design factors of longitudinal smartphone-based health surveys,” J. Healthcare Inform. Res., vol. 1, no. 1, pp. 52–91, 2017. 10.1007/s41666-017-0003-8 PMC8981805 35415393

[74] S. Vhaduri, J. Cho, and K. Meng, “Predicting unreliable response patterns in smartphone health surveys: A case study with the mood survey,” Elsevier Smart Health J., vol. 28, 2023, Art. no. 100398.

[75] S. Vhaduri, A. Ali, M. Sharmin, K. Hovsepian, and S. Kumar, “Estimating drivers' stress from GPS traces,” in Proc. 6th Int. Conf. Automot. User Interfaces Interactive Veh. Appl., 2014, pp. 1–8. 10.1145/2667317.2667335 PMC4390997 25866847

